# Role of gender participation in urban household energy technology for sustainability: a case of Kathmandu

**DOI:** 10.1007/s43621-021-00027-w

**Published:** 2021-03-23

**Authors:** Bindu Shrestha, Sudarshan Tiwari, Sushil Bajracharya, Martina keitsch

**Affiliations:** 1 Tribhuvan University, Kirtipur, Nepal; 2 Norwegian University of Science and Technology, Trondheim, Norway

**Keywords:** Gender participation, Household, Energy, Sustainability

## Abstract

Sustainability has endured as a global topic in terms of quality of life and energy-saving for an equalized system. Households represent one of the most energy-consuming sectors globally and are expected to increase tremendously in the future. Women have higher responsibility in the household energy use in most societies. However, their participation and impacts have been less prioritized in the sustainability concept regarding energy perspective. In contrast, most development studies reveal that women's participation in managing resources can positively impact women and policy management. However, women's active participation and influences on social, economic, and environmental contexts are mostly ignored in energy-related decisions, disregarding women's productive activities. Thus, this study evaluates the gender role in urban household energy in three contexts of economic, social, and environmental aspects of sustainability pillars. This study is exploratory research based on questionnaire survey, interviews, observation, and air quality tests to apprehend appropriate data. The study revealed that the lower-income group uses a larger share of their monthly income for household energy with unclean cooking fuel. It has an impact on low-willingness to participate in new energy technology purchases. The use of electrical appliances and income has a moderate correlation (r = .48). However, Kathmandu urban households are eager to use electric cooking, but urban women have insufficient knowledge, information, and less affordability for new technology. The city's sustainability level is still low (47 scores) from a gender perspective, and it suggests the city needs a long way on the sustainability route. The study concluded that innovative technical interventions and women's financial power are essential, including the subsidy policy to reduce inequality between low and high energy household share variation and elevate gender participation. The gender mainstreaming approach in energy policy can increase women's participation in energy technology to get a clean environment and reduce the nation's financial burden of importing fuel.

**Supplementary Information:**

The online version contains supplementary material available at 10.1007/s43621-021-00027-w.

## Introduction

The importance of energy and gender integration in policy debates has been significant in sustainable development in the last two decades. Most studies acknowledge that women's participation in the energy sector contributes significantly to achieving global energy efficiency goals for sustainable development [1–3]. Household energy consumption in the world accounts for a 35% share of total energy, and household is the most gendered sphere of society in most cultures [4, 5]. Women's primary responsibilities are household chores that apply both in rural and urban societies of most countries such as Nepal. Numerous studies [1, 2, 10] have proved that modern energy services have improved women's socio-economic status with less time and effort involved in households' chores and reducing the health risks associated with current energy practices. Women can play as key drivers of the innovative and inclusive energy sector for a successful clean energy transition. For this, government and industry need to address barriers to gender participation to foster change [7]. Recognizing the importance of the gender dimension in energy policies, the seventh SDG has prioritized proper access to clean and affordable energy as a universal right [8]. The fifth goal emphasized that gender is an inseparable entity in energy justice for sustainability. Energy access must go beyond meeting basic needs to improve quality of life and economic take-off conditions. It emphasizes innovation, sustainable consumption, and justice [2]. IEA has suggested a C3E program focusing on career development and participation since 2010 to harness all talents and close the gender gap for economic and social benefits [7]. It encourages more women's participation in the clean energy sector. Development research shows that increasing women's energy management participation can achieve a win–win situation for women and policy management [9]. Energy justice is hard to achieve without gender justice. However, women are mostly ignored in energy-related decisions and industries, disregarding women's productive activities [8, 9].

Nepal contained a 28 million population with 27 billion of GDP. The Kathmandu city has a high-density population of 20,288 people per square kilometer and 1.4 million dwellers [11]. Still, 19% population lived under the poverty line. Access to electricity has been improving in Nepal, particularly in urban areas, by 94%. The electricity consumption rate has been increasing by approximately 9% per year [12]. The electricity generated in Nepal comes from clean energy—hydropower. However, most of the energy supply is from biofuels and wastes by 9.64 Mtoe [13], as 21 million people still rely on traditional biomass for cooking. Nepal is one of the least energy-consuming countries globally; however, it has the highest energy intensity in South Asia—4.5 times higher than the world average, Nepal’s energy intensity is 1.8 times higher than India or China [12, 13]. In Nepal, the residential sector accounts for the largest energy consumption by 80%, and cooking holds the maximum energy use for 60% of the total energy share [14]. Women are highly involved in household chores, including cooking activities. Kathmandu city—Nepal's capital holds 22% of its total urban population [15] and is ethnically diverse. Energy is substituted using human muscles (somatic energy), particularly in household chores, such as washing clothes, cleaning, and grinding, and women are considered responsible for those household chores.

Historically, both men and women are considered sources of empowerment as a representation of *Shakti*—male (power) and *Prakriti*—female (nature) in Hindu philosophy, particularly in the global South. Similarly, the equality concept can be acknowledged through the example of *Ardhanareshowr* in Hindu doctrine. Women are worshipped as a goddess in Hindu festival, and those rituals are followed to respect women-hood. However, the society still has divisions in a ranking of higher and lower in terms of virgin and widowed. A larger society of Kathmandu is based on the Hindu religion and followed a patriarchal order. Traditionally, men are considered the breadwinner, and women are managers of the house; nonetheless, women also started to earn and work outside, adding more works to women. In those scenarios, women have been working in three different productive sectors: household, child-rearing, and earning outside, and modern energy and technology usage can make it comfortable to manage all sectors.

The present energy transition towards the efficient world through technologies and globalization has increased a concern of energy and environment that has focused on their role of energy consumption, social, and economic factors [16]. It has emphasized that all citizens are part of the energy equation, and women are also becoming visible. Numerous studies [14, 15] show that women can change towards sustainable energy. Women in the North has been actively engaging in energy issues to shape a gender-sensitive movement towards sustainability. For instance, there is an evolving network of women in the energy sector, but major energy decisions continue as a male domain [18]. Cecelski [16] has expressed that women have not necessarily excluded intentionally nor energy-related activities overlooked, but ‘*they are simply defined out of the energy sector*.' Different theoretical approaches have been initiated to realize women's importance in development: welfare approach, women in development, women and development; Gender and development; and effectiveness approach. Those approaches could not properly amalgamate the gender needs in the energy sector. Then gender mainstreaming rises as a paramount process to consider gender as a holistic way to view women in technology. The incorporation of women in technology and innovative processes is expected to positively shift the mainstream towards meeting the poor and equity needs, the South, and women.

**Fig. 1:**
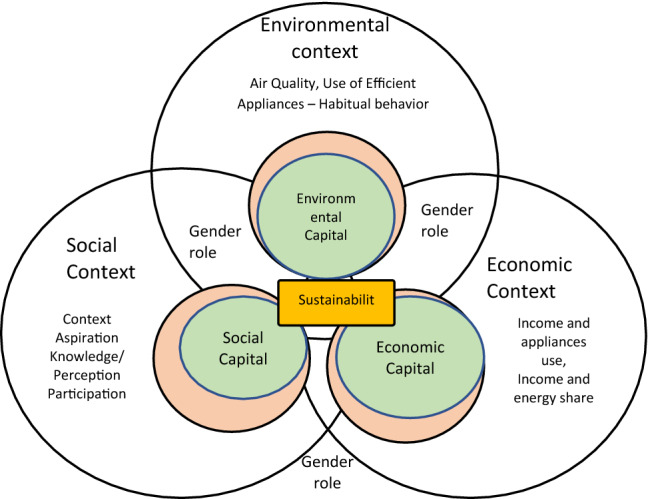
Conceptual framework: sustainability and gender role in energy use (own compile)

A recent study shows that women's energy decisions involvement can reduce the electricity bill up to 23% in female-headed households, supporting the energy-efficiency environment for sustainability [19]. However, the active participation concept is rarely translated into energy-related decisions in the household and policy level of Kathmandu. Kathmandu society is still entrenched in patriarchy that is reflected somehow in women’s participation. Even after the three decades of the Rio summit and Beijing conference of gender advocacy, Nepalese women are still hardly seen in an equal position in the energy sector, even in urban Kathmandu. Half of the population are women in Kathmandu [15], and they are still lagging in exercising their rights in energy decision-making and their active participation. A gap of unequal voices on energy needs [20] and low participation in energy decisions may hinder the sustainable development goals (SDGs) goal achievement. Gender has been aligned concern for sustainability; mainly, social sustainability demands gender equality in every activity [16, 17]. Keeping the gender lens in the energy policy can make it easier to achieve an SDG of 5 and 7. Gender participation in energy consumption plays an essential role in the sustainability path. The gender lens is vital to achieving sustainability goals in terms of energy consumption behavior understanding the economic, environmental, and social contexts of Kathmandu. However, a scant study has been done from this perspective. Thus, this paper first explores the three contexts of energy sustainability, and second, it evaluates the level in three study areas based on identified indicators from the literature and contextual study from a gender perspective. Third, it identifies gender mainstreaming energy policy measures to elevate participation in three sustainability aspects that may help the energy policymakers set long-term policy regulations as shown in Fig. 1.

## Methodology

This study is based on a cross-sectional, descriptive, and explorative study. The research can be categorized into three stages. The first stage: identifying energy use in the households and obtain informed consent from them; the second stage: detailed questionnaire surveys were done and identified different households for interviews and test of household kitchens in three different stratum location; the third stage: comparative and evaluation of sustainability level were done in terms of energy consumption and environmental aspects in a gender perspective. The random stratified sampling survey of Six hundred twenty-three households in sixty neighborhoods was accomplished for diverse respondents, and IAQ tests in the kitchen were done in six distinct households with fifteen extended interviews. The query was raised mainly in three sections of the energy sustainability sector. The economic variables include energy appliances usage and energy share. The social variables include gender participation in energy-related decisions. The environmental variables include kitchen environment data: number of windows, exhaust fan, chimney, and air quality. Onset’s HOBO MX1102 CO_2_ sensor was used for IAQ test in the cooking areas. It has a recording range: 0 to 5000 ppm and accuracy: ± 50 ppm ± 5% on a non-condensing environment. The twenty-seven indicators were recognized from literature and contextual study: five in economic, twelve in social, and ten in environmental sustainability- explored in the three study layers of Kathmandu (Additional file 1: Annex 1.1).

The gathered data were analyzed using SPSS Software, and the qualitative analysis of interviews and observation was analyzed from ATLAS.ti software. The discussion and interpretation were obtained in a neutral voice related to literature. The correlation and cross-tabulation of those variables were employed in SPSS to identify the percentage of sustainability indicators in three study areas. The results identified in percentage and counts were converted into a ten-point scale to obtain a sustainable level based on attitude towards energy-saving and gender participation. For instance, the number of social events may increase in energy—placed on value B. The use of the solar system and efficient appliances uses—value A. The aim of converting a percentage into values was completely based on the researcher's subjective decisions considering energy-saving and gender perspective. The total obtained numerical values were added to reveal the positions' comparison between three city layers as sustainability score as shown in Additional file 1.

### Selection of study area

Kathmandu city is the capital of Nepal—the world's 96th largest country by area. The study areas were identified in inner, middle, and outer-city as three layers based on Kathmandu's urbanization and different socio-economic contexts. The inner-city is mentioned as city layer-1, which history dates to 2000 years old, contained the primary domain of an indigenous group of Newar. The middle-city as city layer-2 that urbanized from the 1980s to 2000s; inhabitants are migrated from the nearby cities and moved from the inner-city. The outer-city, as city layer—3—highly urbanized from the 2000s to the present, and the primary domain of migrants from rural and nearby urban areas, contained mixed ethnicities [19].

Kathmandu city does not have a harsh climatic condition. However, winter season from December to February with an average temperature of 2 °C–12 °C and summer temperature April to September with average temperature is 20 °C–35 °C [21].

## Results

### Economic context and energy consumption

This section studied the economic condition in terms of heating and cooling appliance and household income share (Fig. 1).

#### Electrical heating and cooling appliances in the income group (energy technology use)

**Fig. 2:**
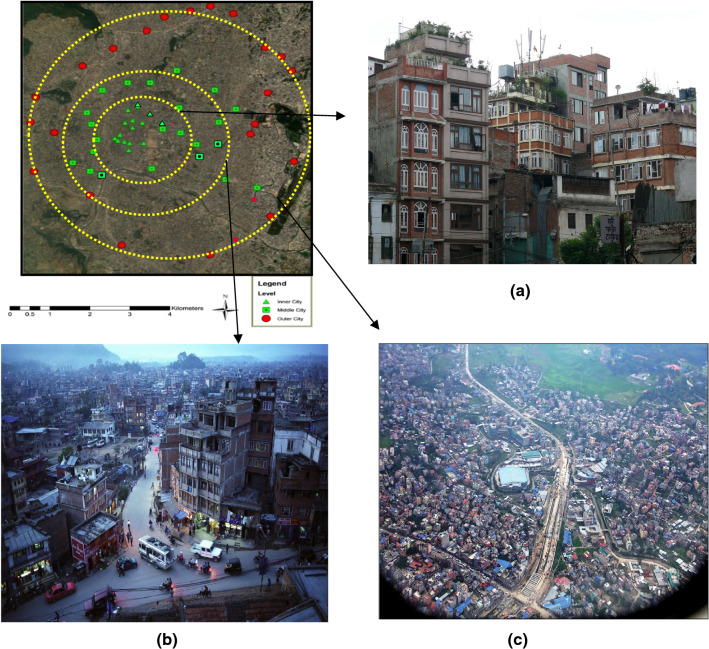
Selection of neighborhoods in three city layers (Author’s compile). **a** Inner-city (Urbanization till the 1980s), **b** Middle-city (Urbanization 1980s–2000s), **c** Outer-city (Urbanization 2000s–2020s)

**Fig. 3:**
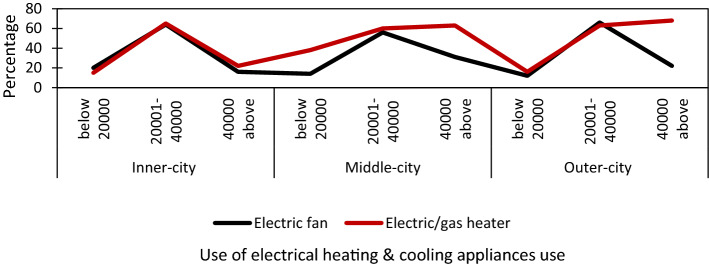
The relation between Heating Appliances and income (Author’s study)

The heating system's data in different expenditure groups demonstrated that the highest use of a heating system of electric and gas heater was found in the high-income group of outer-city dwellers by 68%. The lowest use of electric and gas heater was used in a low-income group of inner-city dwellers by 15%, as shown in Fig. 2. The contrast trend was noticeable in electric/gas heater in the high-income group of inner-city by only 22% that comparatively lower with middle-and outer-city layers (63% and 68%). It might be because the compact settlement pattern in the inner-city resulted in a less cool environment and culture of clothing adjustment. The data of space cooling appliances showed that it was used extensively in all income groups in three city layers (Fig. 2). The highest percentage of electric fans were used (66%) by middle-income respondents of outer-city. The lowest use of the electric fan was found in the low-income group of outer-city by 12%. The surprising trend of using electric appliances was noticeable, with a poor correlation between cooling appliances and income (r = 0.22, p < 0.01). The high-income group of the inner and outer city used moderately less use of electric fans by 16% and 22%. The reason might be because of the new building design with enough ventilations. The results showed that higher income had more significant use of heating appliances, gas and electric heaters, and less natural ventilation. The result revealed that income and heating appliances have a moderate positive correlation (r = 0.48, p < 0.01), the higher the income and high use of appliances (Fig. 3).

#### Income and energy share in urban households

**Fig. 4:**
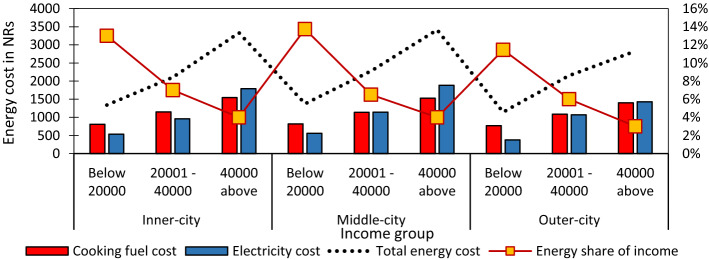
The proportion of income and energy share in three city layers

The indicator demonstrated that the affordability of energy uses amplified disparity in the city. Comparing the proportion of income and energy cost share in three city layers, low-income groups of inner and middle-city spent 13% and 14% of their income while the same group of outer-city spent only 11% (Fig. 4).

The middle-income households spent an energy cost of 6–7% of their income in three city layers. The high-income group of middle-and outer cities' energy share of total income is lower than other city layers (3%). The reason for it is that a higher percentage of high-income groups dwelled in outer city layers. It implied that the higher the earnings, they can afford higher the energy appliances. The energy costs as a proportion of income are substantially higher, directly linked to low-income households' low willingness to spend money on electrical appliances purchase.

### The social context of energy consumption from gender perspective

In this section, social sustainability indicators elaborate on a contextual way of life, customs, values, aspirations, knowledge-perception, and men and women's participation in energy decisions. The twelve different individual indicators are analyzed under four categorized social indicators.

#### The tendency of shifting energy technology and adaption

**Fig. 5:**
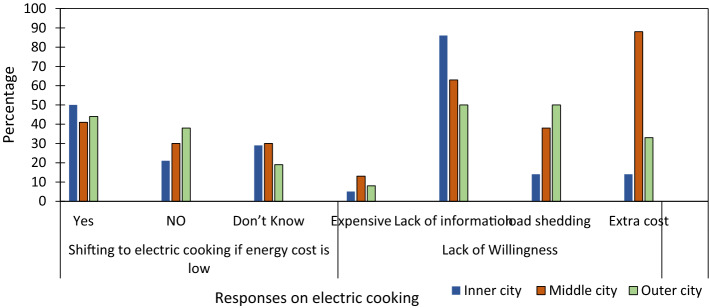
Respondents views on the electric cooking system

The trend of shifting towards cleaner fuel with new technology could resolve the fuel crisis. Respondents were asked about their interests in changing new technology for the cooking system if energy cost was reduced compared to the existing one. A higher percentage (50%) of inner-city (middle-city—41%, outer city—44%) respondents were eager to shift to the electric cooking system. In contrast, a quarter of respondents were confused and reluctant to use it due to various reasons. It underlined majorly two significant reasons. Firstly, a lack of information about the electric cooking system could not increase interest in dwellers, and secondly, dwellers could not afford extra utensils needed for the new cooking system. For instance, 86% of inner-city respondents showed unawareness, and 88% of middle-city respondents expressed unreliability and unaffordability on the technology due to the extra cost of specific utensils. 50% of respondents had a fear of frequent power shortage in the city as unwillingness. However, electricity has been a continuous supply in the city since 2017 (Fig. 5). Observations and interviews showed that an induction stove available on the market had a single burner that made it challenging to cook varieties of food in a short time. The result implied that respondents had a low willingness to shift towards electric cooking due to fear of affordability, accessibility, proper information gap, and design issues and remained to use LPG as cooking fuel.

#### Knowledge and perception on energy technology 

**Fig. 6:**
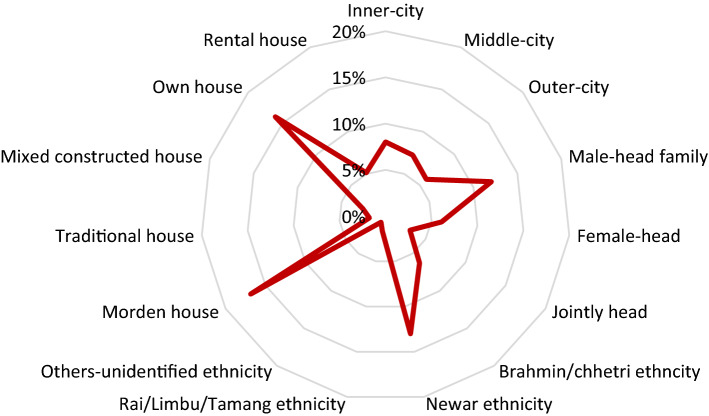
Rainwater harvesting in different variables

The knowledge of clean energy use and efficiency practice has a significant role in achieving social sustainability. The rainwater harvesting trend was higher in the Newar group (13%), inner-city dwellers (8%), headed family (12%), modern houses (17%) living in their own house (16%) (Fig. 6). The lowest rainwater harvesting was found in rental dwellers (5%), traditional houses (2%), and other unidentified ethnicities. The reason for it might be that unidentified people were migrants living in rental spaces, did not have built a rainwater collection system in the building, and lived in a single room without a terrace. It showed that knowledge and practice on rainwater uses were higher, particularly in Newar ethnicity, male-headed families, nuclear family composition, and self-owned dwellers. It revealed that residency type, headship, and building design construction method influenced the rainwater harvesting trend and behavior.

#### Participation in energy technology decisions

**Fig. 7:**
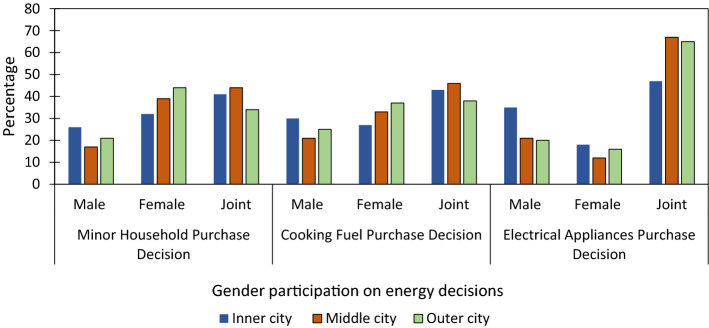
Decision participation in cooking fuel purchase—influences of various variables

In the participation of minor household decisions, joint decisions scored a higher percentage in inner-and middle-city by 41% and 44%, respectively. At the same time, female decisions were increased by 44% in middle-city households. The increased number of females in household decisions as jointly and singly implied that increasing participation and leading quality in decisions increased. The electrical appliances and cooking purchase decisions were increased in three city layers by 47% to 67% and 38% to 43% (Fig. 7). The result showed that urban households had a trend of joint decisions in most of the household decisions.

### Environment context for energy consumption  from gender perspective

The environment context of urban households is described as the kitchen environment in terms of ventilation, electric kitchen hoods to reveal the air quality of space. The study showed that higher energy consumption occurred in the household for cooking activities. Thus, the kitchen was taken as a significant study place in the household in this study.

#### Use of electric kitchen hoods and ventilations in urban kitchens considering gender impacts

**Fig. 8:**
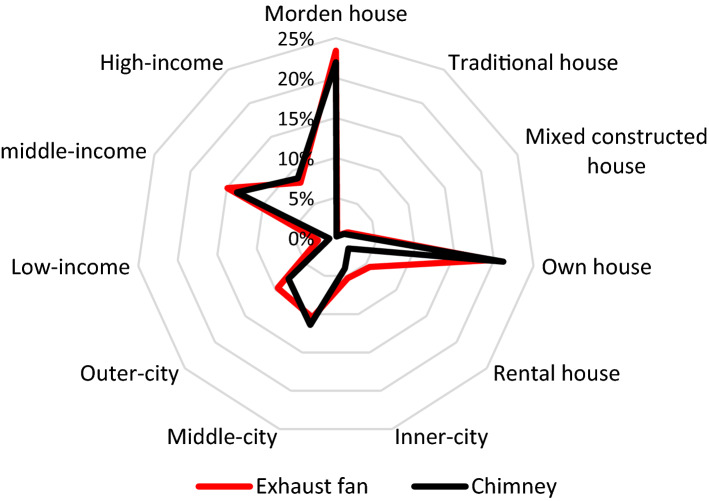
Use of exhaust fans and chimney in various building types

Exhaust fans and chimneys were higher in modern buildings than traditional and mixed buildings (traditional structures with concrete finishing). The data showed that 23% of modern buildings contained exhaust fans, and 22% consisted of chimneys, while the traditional building contained exhaust fans only by 0.3% of chimneys by 0.16%. Mixed buildings contained 2% of exhaust fans and 1% of chimneys (Fig. 8). It showed that the electric kitchen hoods used to evacuate cooking smoke were higher in modern buildings than traditional ones. The observation indicated that the newest buildings consisted of kitchen hoods and ventilation for a healthy indoor environment.

The inner-city respondents used exhaust fans by 5% and chimneys by 4%, respectively. The middle-city respondents used exhaust fans by 10% and chimneys by 11%, respectively. Similarly, outer-city respondents placed exhaust fans and chimneys by 10% and 11%, respectively, as shown in Fig. 8. Overall, the use of exhaust fans and chimneys in the kitchen was higher in the owned household by 20% and 21% compared to rental households' kitchens. However, natural ventilation is indispensable in cooking space for the cook's comfort and to maintain hygiene. The results showed that urban kitchens without windows were found by 37%, in inner-city, and having a single-window by 71%. The test of differences was statistically significant between location with kitchen hood use and ventilation in the kitchen (x^2^ = 20.66, p < 0.01, r = 0.182 and x^2^ = 41.18, p < 0.01, r = 0.25) having a low effect.

#### Kitchen environment from gender perspective

**Fig. 9:**
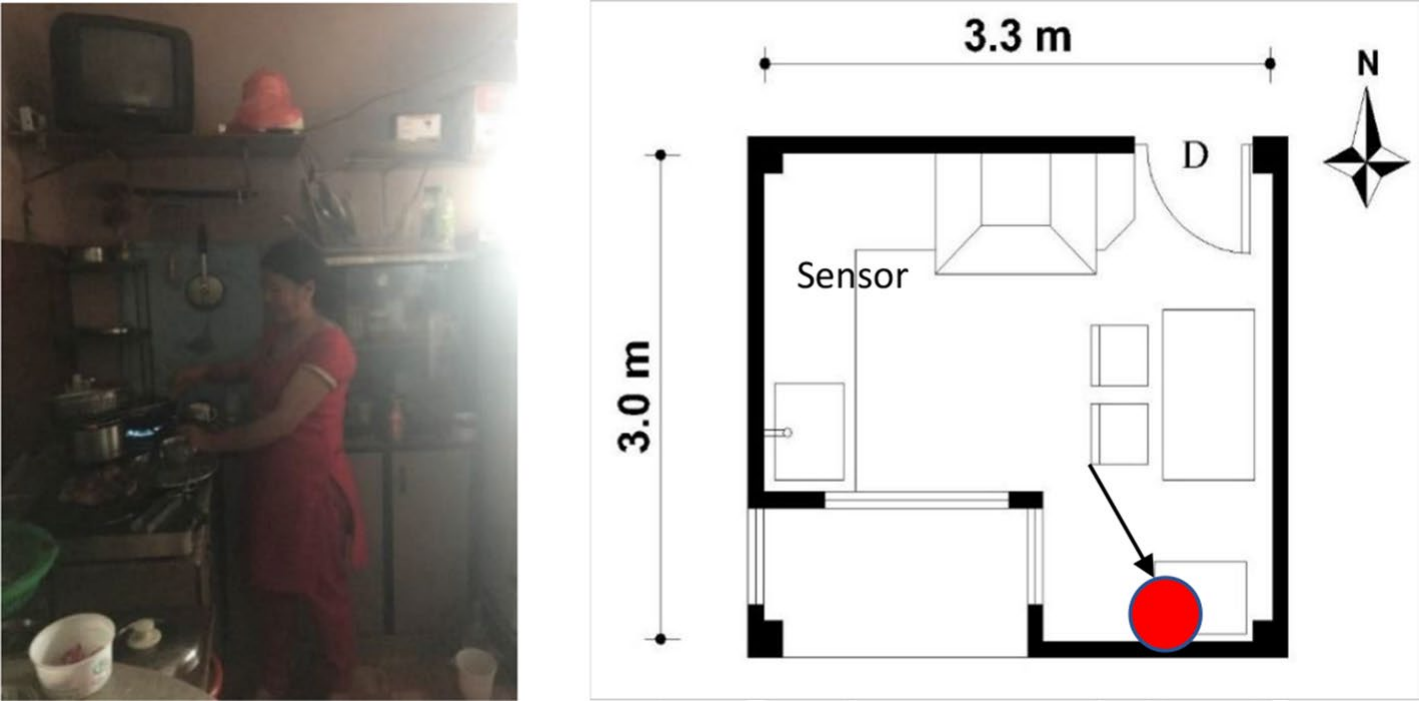
Respondent cooking in the kitchen D3 (own study)

**Fig. 10:**
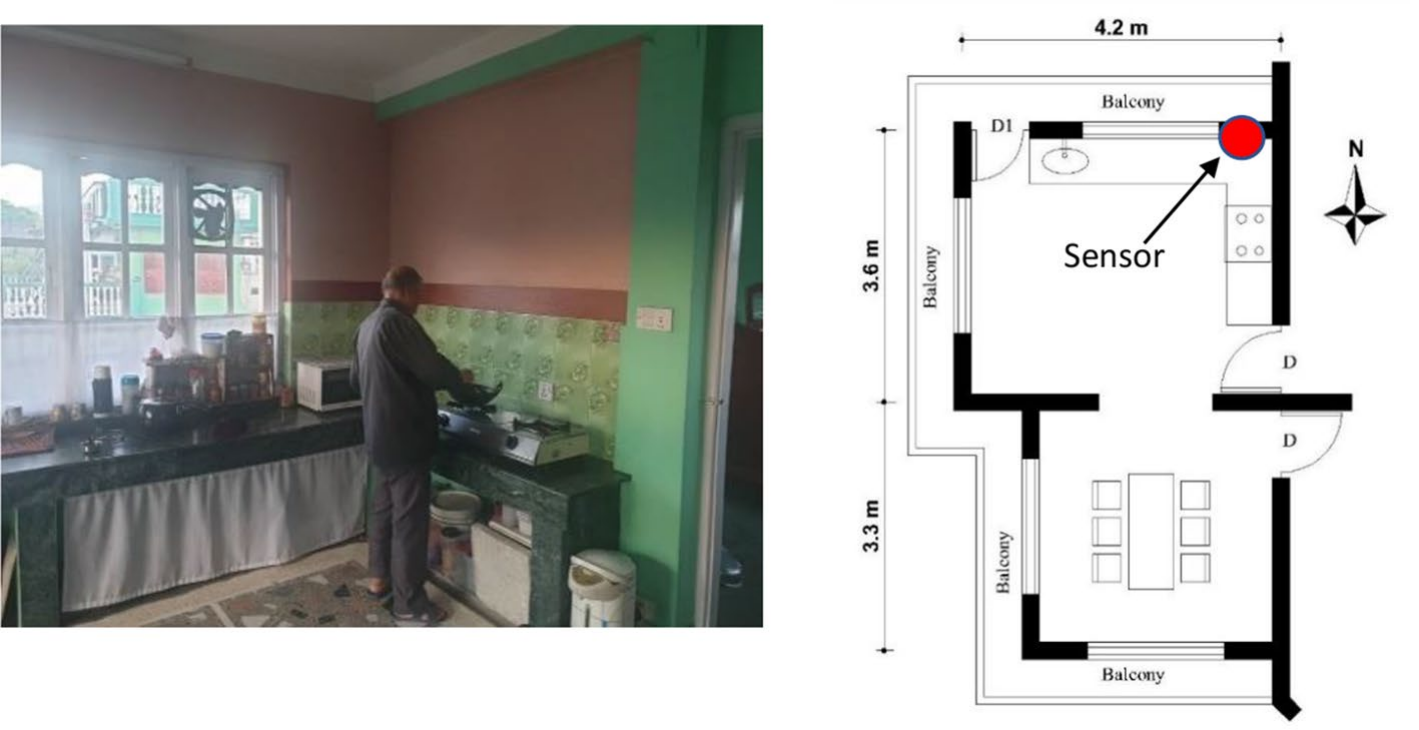
Respondent cooking and plan of D2 household (own study)

WHO standards and American Society of Conditioning Engineers (ASHRAE) has provided ventilation standards to maintain IAQ from CO_2_ concentration level and suggested need of windows open for fresh air flow and healthy air quality. The inner-city study households' cooking spaces were found as combined with a dining area with a window. The study revealed that the kitchen with a self-owned household had a ventilation provision in the kitchen, while rental spaces were found without ventilation nearby the cooking area. Few kitchens were found to have exhaust fans, but chimneys were not found. For instance, the D12 household kitchen (Table 1) had 9.6 m^2^ space without an exhaust fan and chimney but have a combined door-window on the cooking space's rear side. Simultaneously, rental spaces (D8 & D13) did not have windows and chimneys in the cooking spaces, as shown in Figs. 9 and 10.

**Table 1:** Detailed data of air quality in the urban kitchen (Own compile)

Households	Area (m^2^)	Family members	Samples	Date	Kitchen design	Temperature	Relative humidity	Air quality—CO_2_ (ppm)
D12	9.6	3	1428	1/31/19 to 02/15/2019	No kitchen hood, exhaust fan, improper position of the ventilation	Temp.− 10 °C–23 °C	70%	Min.—367 Max—3683 Avg.—603, St. dev.—241
D13	6.3	1	2344	02/15/19 to 02/23/19	None of the ventilation, exhaust fan, & chimney	Temp.− 13 C–18C, Avg. 14 C	77%	Min. 291 Max. 2239 Avg. 703 St. Dev. 480
D8	5	3	2330	02/24/19 to 03/04/19	No ventilation but consist of exhaust fan	Temp.− 15 °C–20 °C, Avg. 17 °C	60%	Min. 343 Max. 2521 Avg. 519 St. Dev. 314
D3	7.2	4	2890	4/10/2019 to 04/20/2019	Consist of exhaust fan, chimneys, proper ventilation	Temp. Avg. 25 °C	59%	Min. 340 Max. 2266 Avg. 623 St. Dev. 341
D2	14	5	7510	12/07/19 to 01/02/20	Cross ventilation and exhaust fan	Temp. Avg. 13 °C	68%	Min. 331 Max. 1981 Avg. 647 St. Dev. 363
D6	14	5	4583	01/10/20 to 01/25/20	Cross ventilation and modern chimney	Temp.− 11 °C, 9 °C–16 °C	75%	Min.202 Max. 1782 Avg. 591 St. Dev. 379

The middle-and- outer city case studied houses showed that the size of kitchen & dining was more extensive in the self-owned houses. For instance, D2, D3, and D6 houses had 14, 7.2, and 14 m^2^, respectively, with two windows, exhaust fans, and chimneys (Figs. 9 and 10, Table 1). It revealed that most of the self-owned household kitchens have ventilation while rental spaces lacked ventilation and an electric kitchen hood. It illustrated that most rental kitchens did not have consideration of the cooking environment.

#### Air quality of urban kitchen

The cooking culture also impacts on air quality of the kitchen. The households D13, D12, D3, D2, D8, and D6, had a maximum air quality in terms of CO_2_ level were above 1000 ppm, and the highest was noted on D12 that was 3683 ppm, and the average level was 603 ppm (Table 1). However, this household had minimum cooking food items but had a high CO_2_ level was noted. It might be because of an improper ventilation position on the cooking area, lack of kitchen hood, and exhaust fan. Similarly, the maximum CO_2_ level was found during cooking in the D13 household was 2239 ppm, and the average level was 703 ppm (Table 1). This level was surprisingly high for a single-family and cooking culture with 2–3 items. The reason can be noted that the cooking area did not have any ventilation, exhaust fan, and chimney to evacuate smoke that resulted in poor air quality in the room, as shown in Tables 1.

**Fig. 11:**
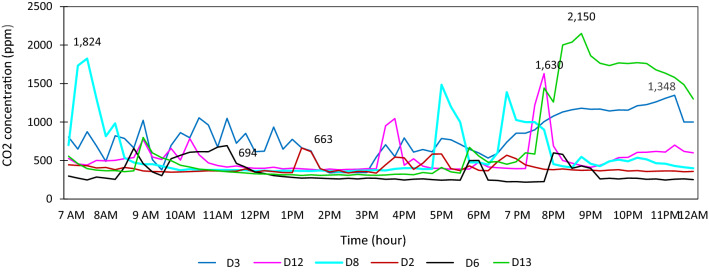
CO_2_ concentration level in the urban kitchen (own tested)

Table 1 showed that the CO_2_ concentration level was much higher in all households. The samples were taken for 7–20 days in each household, and it did not reveal actual IAQ in the kitchen area to evaluate reasons for high CO_2_ concentration. To evaluate and identify reasons for IAQ and the role of ventilation behavior in a kitchen was observed for a single day for 24 h on a regular routine in those six households. The comparison of the households' CO_2_ concentration level could illustrate the role of ventilation and kitchen hoods appropriately in IAQ, as shown in Fig. 11.

In the D12 household, cooking dinner time was higher than morning time, and CO_2_ concentration raised to 1630 ppm with only LPG for cooking. The trend of average ppm remained below 1000 ppm. The kitchen did not have any exhaust fan and chimney, but openings were located rear side of the cooking area. The average CO_2_ concentration level was 523, with a variance of 209. In the D13 household, CO_2_ concentration level in a day was higher in evening dinner cooking time, raised to 2150 ppm. The cooking space did not have ventilation, exhaust fan, and kitchen hood. The average CO_2_ concentration was 728 ppm, with a variance of 589. A higher variance was noticed in this household, as shown in Fig. 11. In the D8 household, this household's female used to cook early morning to manage the office and children's early school. After coming back office, she prepared snacks in the evening and prepared dinner at 7 PM around as shown in Fig. 11. The highest concentration level of CO_2_ was found in the morning, and evening levels were also relatively higher above 1000 ppm.

The distinct increase of CO_2_ level was linked to the lack of ventilation and kitchen hoods; however, the exhaust fan was seen above the cooking area (Fig. 10). The average CO_2_ concentration level was 574 ppm, with a variance of 343. D6 household kitchen consists of proper cross ventilation near the cooking area and on the rear side, including kitchen hood at the top. Besides, the fuel used was LPG and induction. The average CO_2_ concentration was 341 ppm, with a variance of 133. It was lower than other households. The D2 household demonstrated relatively low CO_2_ concentration levels even in the morning and evening. The highest concentration of CO_2_ in a day was 663 ppm in the afternoon (snacks time and average during 3–5 PM). The kitchen had large openings on three sides with an exhaust fan at the top of the cooking area. The family's food culture was simple, with less frying, grilling, and less cooking in a day. The average CO_2_ concentration was 574 ppm, with a variance of 73. In the D3 household, the kitchen was mostly used the whole day due to making tea and different family members' eating schedules. However, the maximum CO2 concentration was found at nearly midnight (1348 ppm) due to the habit of taking late-night dinner. The kitchen had an exhaust fan and ventilation; however, it was not near the cooking space. The mean CO_2_ concentration was 802, with a variance of 266. In all households, the CO_2_ concentration level was lower than 1000 ppm on a regular day, as per the ASHRAE standard. The least was found in D3 with 341 ppm and highest in D6 and D13 (802 ppm and 728 ppm).

#### Air quality on social events and gender role

Family & friends gathering and cooking varieties of food in the social events are part of energy-consuming activities. The Kathmandu city consisted of inherent cultural and religious beliefs. Respondent of inner-city—D12 household had less frequency of social activities in the home and low household energy consumption.

**Fig. 12:**
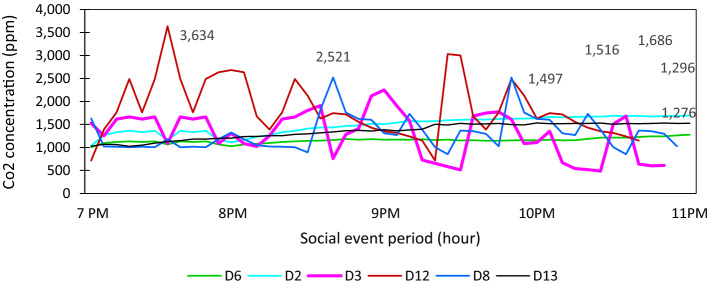
CO_2_ concentration level in the urban kitchen during social events (self-tested)

However, IAQ in terms of CO_2_ concentration level resulted higher during a social gathering, raised to 3634 ppm as shown in Fig. 12. D8, D3, and D6 households used to gather more than 20 numbers within a year, and D2 hosted 10–20 household events. CO_2_ concentration level of D8 increased on 2521 ppm, 2250 ppm in D3, 1296 ppm in D6 households. Besides, females of those households spent almost 3–6 h in the kitchen every day to prepare, cook, and clean the kitchen. The D2 household respondents were retired who had a regular monthly income and shared household chores equally with average social gatherings. CO_2_ concentration level of D2 increased to 1686 ppm maximum. The cooking culture of this household was based on religious philosophy. Similarly, the household D6 family members were working outside and helped each other in the household chores. However, this family had a hierarchy of gender roles to some extent, for instance, energy decisions. The D13 household host rarely social events, but during friends gathering, the CO_2_ level measured maximum was 1516 ppm, as shown in Fig. 12. The observations showed that low-income households living in rental spaces used fewer electrical appliances and less social activities. However, those spaces had relatively higher CO_2_ levels compared to household owner's space. One of the contributing factors to an increased level of CO_2_ in the Kathmandu urban kitchen was found as a social gathering holding several occupancies. Shen et al. [22] and Taneja et al. [23] underlined that cooking activities contributed to emitting CO_2_, including other pollutants. Additionally, the trend of increased indoor CO_2_ level was higher in winter compared to summer. In this regard, factors included indoor activities, closed ventilation, and human occupancy duration influence in increasing CO_2_ concentration due to limited floor area and human metabolism. However, information on IAQ in the kitchens in urban households was minimal in Kathmandu. In this study, females cooked most of the time and were exposed to emission in lack of proper ventilation. However, women were less aware of it, and eye irritation and mild respiration were felt for a specific cooking time, but it was considered normal. It showed that women, including the remaining family members, were less aware of the IAQ.

#### Cooking culture

The observation on cooking showed that it also impacts energy use patterns and air quality of the kitchen. The respondents of households D12, D13 cooked 2–3 items daily while D8, D3, D2, and D6 cooked 4–6 six food items daily for two-time meals. D12 residents had food habits based on fruits and salads on the main meal course compared to cooked vegetables due to less time to cook. The daily energy-intensive activities were shallow due to food habits, but the maximum using fuel was for boiling water for daily drinking and bathing. In contrast, the D13 household's respondent was single, so cooked fewer items on the main course and ate day snacks outside, resulting in less energy consumption. On the contrary, household D2 cooked a similar number because of being vegetarian, and the family was based on religious belief and self-energy consciousness. The result showed that health issues influenced cooking culture, food habits, availability of cooking time, religious and cultural aspects that ultimately influenced energy consumption to a certain extent and influence on IAQ of the kitchen. However, this study could not provide enough statistical data on the relationship.

The CO_2_ concentrations in urban kitchen gradually increased during cooking but decreased whenever the residents used natural ventilation. When only the kitchen hood was used, the level of CO_2_ concentration decreased slightly compared to the levels with no ventilation. It revealed that natural ventilation could rapidly improve IAQ by reducing CO_2_ concentration than did mechanical ventilation. This result was in line with Lee et al. [24] and Batog and Badura [25]. Besides, Pokharel and Rijal [26] emphasized that poor ventilation ultimately increased CO_2_ concentrations, resulting in poor IAQ, and recognized that building characteristics, such as infiltration and ventilation rate, play an essential role the variation of indoor CO_2_ concentration. Further, Lee et al. [24] and Rahman and Islam [27] investigated that kitchen hood systems can reduce CO_2_ concentration (@ 1.5 m/s hood system Vs. close vent) by ten times by the use of an efficient hood system to maintain thermal comfort inside the kitchen space.

### Sustainability level from gender perspective in terms of energy technology

After identifying three sustainability pillars findings—economic, social, and environmental contexts in three city layers, the sustainability level had evaluated based on their activities either towards moving energy efficient or not. In comparing three study city-layers, it was found that electric fan use was higher in the inner-city. The use of an electric heater, vacuum cleaner, and solar use was higher in the middle-city. The sustainability indicator was taken as a checklist to compare three city layers and evaluate the city in sustainability based on local household energy data from a gender perspective. The results showed that middle-city used higher energy use, as shown in Additional file 1. The middle-and outer-city started using efficient appliances use, clean energy, and assisted in achieving the economic sustainability value of 45 (Additional file 1: Annex 1.1). Similarly, inner-city was prevalent in cultural activities and fuel stacking nature keeping extra cylinders. The female participation in electrical appliances purchase (EAP) is higher compared to middle-and outer city layers. In contrast, joint participation in EAP and clean cooking were higher in the middle-city layer than in the rest areas. Meanwhile, the awareness and knowledge of rainwater harvesting and female participation in cooking fuel purchase (CFP) were higher in the outer-city. It resulted in the middle-and outer-city having gained social sustainability values of 55 and 52, and the inner-city achieved 47 (Additional file 1: Annex 1.2).

It was apparent that the cooking culture influences the air quality of the kitchen. The ventilated kitchen, use of exhaust fans/chimneys were high in the middle-city. While comfort feeling during cooking and clean energy use were expressed in higher among outer-city dwellers, however, the presence of windowless kitchen at the same time. The environmental sustainability showed that the outer-and middle-city households received 51 & 48 and inner-city achieved 39 (Additional file 1: Annex 1.3). The low sustainability score of the inner-city was due to high cooking culture and a lack of ventilation and kitchen hoods. The overall score of economic, environmental, and social aspects of inner-city epitomize low energy sustainability (42 points), outer-city (51) on average, and middle-city (48) has ranked higher among three (Additional file 1). This study shows that the city's overall energy sustainability from a gender perspective acquire 47 points combining three aspects.

**Fig. 13:**
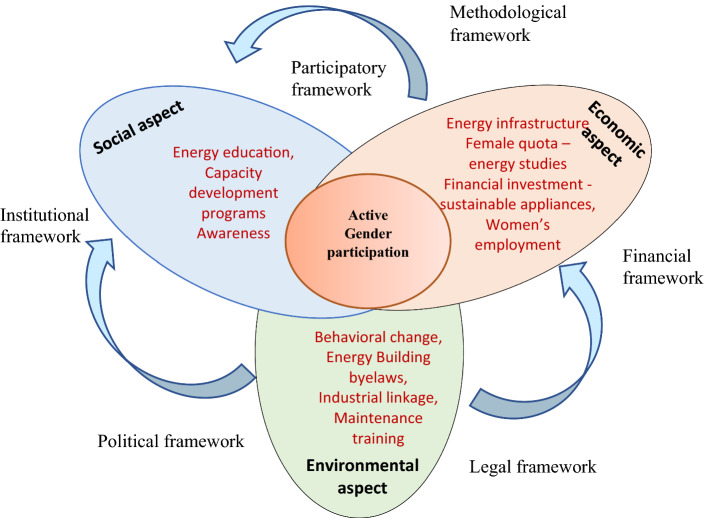
Recommended sustainable energy policy for maximizing gender participation (Own compile)

The criteria were identified from the literature and refined based on study observation and interviews for the context-based sustainability of energy consumption. Three contexts of sustainability were interconnected and enhanced by the primary users of energy. Women acted as agents to enrich the economic, social, and environmental capital, as shown in Fig. 13. Women had a crucial role in increasing energy productivity and enhancing economic, social, and environmental sustainability, influencing sustainability indicators. The three contexts of sustainability were interlinked to each other in terms of gender norms and energy-saving behavior.

### Discussions and policy considerations for gender participation

As asserted by Gatersleben [28], Masera et al. [29], Bisu et al. [30], and Muller & Yan [31], multiple fuel usage does not always ensure awareness about energy-saving, as residents were compelled to use mixed fuels and resulted in the fuel stacking model in three city layers. It created a social gap between rich and poor within the same neighborhood due to the corrupted market. Consistency with Barr et al. [4], Gatersleben [28], and Lutzenhiser [32], social sustainability is strongly based on culture and practice. In urban Kathmandu, electrical appliances had been increasing extensively, particularly in the middle- and higher-income groups, for instance, electric heater and fans, but it was used consciously for limited hours. The natural ventilation and wearing clothes in layers were practiced as an adaptation model in Kathmandu for extreme weather. It demonstrated that culture and belief are still entrenched in Kathmandu urban dwellers in energy use and saving practice, and it can be understood through residents' voices as followed.

*“I used an electric heater just after bathing in winter for half an hour only, but my husband and daughter stuck on a whole day of winter. I have to work continuously in the household, so not possible to sit near the heater.”*

:#D8 & D10 household respondents

Energy use (heating and cooling appliances) is positively related to households' thermal environment and people's thermal comfort—the households of inner-city in Kathmandu holding compact settlements of traditional and modern buildings. As evidence, Rijal et al. [33] and Rijal [34] investigated that traditional houses had high-temperature control in summer and winter due to local traditional material, opening size, clothing adjustment, and food culture. Besides, Gautam et al. [35] investigated thermal acceptability between local and migrant peoples and identified that local people appear more tolerant and saving behavior than migrants due to physio-psychological expectation. This evidence was highly matched to the results of Shrestha et al. [19]. The outer-city residents had higher use of heating and cooling systems than the inner-city, and the inner-city was predominate of local people. Aligned with Davis [36], Gatersleben & Vlek [37], Levett [38], and Nasir et al. [39], ownership demonstrated a significant role in energy uses and saving behaviors. The results showed that joint decisions were higher in all categories except in female-headed families. Female-headed and low expenditure households had higher involvement of females in energy purchase decisions. It revealed that when the women had financial power in hand, they could decide what they wanted for the home with increased active participation, and it can be well explained from the resident's voice as followed.

*“My husband gave me very little money. I did not go outside because I did not have money. I used to buy very little and if finished again ask for money from a husband. In that case, he used to be angry, so I did not buy anything for me in those two years”.*

: #D4 female respondent

Even women from the high-income group had expressed less participation in purchasing and using new technology due to their fears and lack of basic technical knowledge that was influenced by upbringing roots. At present, media and social networking had helped women to get information about the technology. Still, lack of own income and proper education had limited them to exercise their participation in energy technology. It can be well understood from residents’ voices as followed.

"Even availability of advanced technology if he/she is not educated, they will be afraid to use and may have difficulties understanding the system.

# D1, D4, D5, & D6, male respondents

“Media—TVs also helped to teach the system of new technology and comfortability.”

# D1, D4, D9, female respondents

Clancy et al. [1], Habtezion [2], Oberhauser [6], and Gatersleben [28] claim that modern energy services with electric appliances have improved women's socio-economic status by reducing the time and effort involved in households' chore, and this is also the case in Kathmandu. The increasing use of electrical appliances was reinforced to reduce urban drudgery and enhance new kitchen culinary recipes in a limited time. Besides, men also started to help with kitchen chores. Besides, environmentally, kitchen design and culture had been improved due to electric kitchen hoods for air quality. In contrast, the air quality results showed the inner-city kitchen environment, especially in the rental spaces lacking ventilation, exhaust fans, and chimneys. Kurmi et al. [40] studied the 24-h respirable dust concentrations on urban kitchens in Kathmandu identified the presence of 30–100 lg m^3^ dust particles even using LPG cooking fuel. However, it was considered of 57% efficiency. Lee et al. [24] and Aberilla et al. [41] identified that electric cooking has high energy efficiency (80%) than LPG and CO_2_ concentration was less in the kitchen using an induction stove because appliances emit lower levels of contaminants, and it is recommended to improve IAQ. Simultaneously, few studies showed that the rooms' poor air quality or increased CO_2_ concentrations adversely impacts decision-making performance. Fisk et al.'s [42] study showed that people who stayed below 600 ppm have higher decision-making capacities. People living with less ventilation demonstrated health problems frequently. In particular, those conditions might have affected more in the current pandemic context COVID-19 [43]. The urban kitchen's indoor air quality was often overlooked and women's cooking hours enlarged during the COVID-19 pandemic. Cooking oil, smoking, and human activities are essential contributors to indoor air pollution (IAP) due to fuel emissions, people's smoking, and cleaning home [44]. It is suggested that urban housing, particularly kitchens, need to be improved in the context of the COVID-19 to reduce infectious diseases and improve indoor air quality. It can be progressed from proper cross ventilation, natural light (glazing, skylights, orientation), reducing CO_2_ concentration level as a solution that also increased sustainability of buildings' bioclimatic performance. It contributes indirectly to the fight against COVID-19 by improving health conditions over a large timescale of more than 25 years [36, 38, 39].

Sustainable development 2019 report showed that Nepal was ranked in 103 positions with 68 SD scores of seventeen SDGs [45]. In comparison, this study of Kathmandu city obtained only 47 scores. The low score was obtained due to the differences in indicators as this study has developed the indicators based on a comprehensive combination of SDGs 5 and 7 integrated manner. It may not be directly comparable due to differences in indicators set-up. However, it reveals that energy accessibility and gender equality are still challenging for both the city and national context in combined results. Whereas the individual study may result in the piecemeal approach and combined approach can influence the energy just in a real sense with gender justice. Besides, most researchers [14, 39] and government data showed that Nepalese households used electricity, only 50 MJ/household/monthly or 90–100 kWh that is relatively lower than in developing and developed countries. It is worthy of using less energy. However, social sustainability suggested that it is equally essential of a certain standard of quality of life, as discussed by Gatersleben and Vlek [37], Carrera and Mack [46], and Santoyo-Castelazo and Azapagic [47]. It is essential to balance quality of life using energy-efficient appliances in the households holding habitual saving behavior. It implies improving overall comfort to women so that they can participate in economic development.

The economic sustainability of Kathmandu revealed that high-income residents spent more than low-income dwellers, but the energy share of households of total income was low due to high-income affordability power. These conditions are perceived in most developing and in-lined with research by Harris [48] and Santoyo-Castelazo and Azapagic [47]. It resulted that women of the low-income group had a low willingness to participate in electrical appliance purchases due to low affordability. It generated a disparity in society when there was no subsidy for low-income that continues a vicious cycle creating a gap of energy share. The use of appliances in all city layers is low. However, it has been increasing rather than in the last decades, but women still have lower technical knowledge.

It is evident from the results that women do cooking and most household chores in Kathmandu city. Still, they are less involved in technology-related decisions, then the only improvement of cooking practices and space (electricity, ventilation) will be ineffective. Moreover, ethnicity and gender have influenced household decision-making; thus, it suggests mainstreaming gender with considerations of different ethnicities and income groups. The study suggested that gender mainstreaming (GM) intervention should account for gender concerns both at the micro and macro levels in terms of increasing participation of women in the decision-making process, planning to the implementation stages [49]. Practically, it is essential to consider energy-saving and reduce women's labor and strategic gender needs, providing opportunities for women to be involved in social and economic activities for self-enhancement. All urban people still could not afford to have enough facilities, which means urban people are still not equipped with sustainable technologies. Thus, it is suggested to enhance GM strategy of an energy policy based on increasing active participation. When people do not have enough knowledge, they might be realized only after applying the technology. For this, the government must initiate and intend to improve the city's energy situation.

To improve sustainability, it is essential to balance three major aspects of social, economic, and the environment with active participation in energy-related technology purchase decisions. As shown in Fig. 13, this study emphasizes women's energy education, capacity development, and awareness program to improve urban women's social aspects. The investment in energy infrastructure, subsidy on efficient appliances, and increment in women's employment can actively enhance urban women's economic condition to participate in energy technology decisions. Similarly, improvement in urban building regulations in terms of energy concept, training for women on maintenance, industrial linkage development is suggested with a behavioral change in urban dwellers about the IAQ. All three contexts are interlinked in terms of the methodological, financial, legal, political, and institutional framework of gender mainstreaming. Thus, those frameworks should be strengthened from the governmental level and concerned stakeholders.

### Conclusions

The result of sustainability conditions about energy usage concludes that there is a moderate positive correlation between energy consumption and income (r = 0.48). The lower-income group uses a larger energy share of their monthly income and depended on unclean cooking with a less healthy kitchen lacking a chimney, exhaust fan, and ventilation. The issue of IAQ is complex and requires an interdisciplinary study to address them by the house owner, consultant, and government regulations to optimize the quality of indoor air. The high energy share in low-income groups directly impacts a low willingness on energy technology purchases. However, female participation has been increasing in joint decision patterns on energy decisions. Females are still less vocalized in technology-related decisions. It suggests that women should be encouraged to participate in the technology movement and proper information dissemination for integrated energy policy to reduce gender inequality, maximizing clean energy for a better quality of life. The government sector must have a political commitment to ensure equal participation of men and women to seek their equal voices in the planning, design, and implementation stages. Energy-related organizations should commit the community and neighborhood to disseminate technical knowledge and skill development programs as needed. The sustainability perspective suggests to gain energy education, behavior change on inhabitants, improving urban building bye-laws from energy/environmental aspects, Financial investment for women, and sustainable appliances, and hints the linkage of industrial linkage and dwellers for the needed cooking stove designs.

Conclusively, gender and energy have a broader impact on sustainability than current research has shown so far, and it should be further investigated in different related variables. The study is not enough to conclude the sustainability goal only based on a single city study. It demands the study of numerous cities to be studied based on combining two SDGs goals of gender and energy in an integrated manner for conclusive results. This study is more relevant for the energy policymakers and industrialists to design policy and measures holistically. This research can be viewed as the departure point for exploring energy-related decisions from a gender perspective. In current practice, the gender lens is missing in the energy policy development, including household levels resulting in an inefficient energy policy and an inequitable society. The knowledge of social, economic, and environmental scenarios on household energy consumption has supported recognizing contextual sustainability in energy and gender on the same footing. It makes it possible for energy planners and policymakers to have a vision on the present and future scenario to upgrade the quality of life managing energy systems maximizing equal gender participation in the technology movement.

## Supplementary Information

**Additional file 1.** Additional tables.

## Data Availability

The authors declare that all data supporting this study's findings are available within the article with additional information file.

## Abbreviations

CFP: Cooking fuel purchase
C3E: Clean energy, education, and empowerment
GDP: Gross domestic product
EAP: Electrical appliances purchase
GM: Gender mainstreaming
IEA: International Energy Agency
IAQ: Indoor air quality
SDGs: Sustainable development goals
SPSS: Statistical Package for the Social Sciences
