# Supplementary material for: Role of gender participation in urban household energy technology for sustainability: a case of Kathmandu
Source: Discov Sustain. 2021 Mar 23;2(1):19. doi: 10.1007/s43621-021-00027-w (PMC7985919; doi:10.1007/s43621-021-00027-w)
Supplement: Supplementary file 1 — Additional file 1. Additional tables. [file 43621_2021_27_MOESM1_ESM.docx]

**Role of Gender Participation in Urban Household Energy Technology for Sustainability: A Case of Kathmandu**

**Annex**

**Annex 1**

Annex 1.1. Economic sustainability indicator and checklist

| **Criteria** | **Description** | **Inner-city** | **Middle-city** | **Outer-city** | **Description** | **Values** | **Inner-city** | **Middle-city** | **Outer-city** |
| --- | --- | --- | --- | --- | --- | --- | --- | --- | --- |
| **Income and number of energy use** | Proportion of income and electrical appliances use | Low-income-7 (24%)  Middle-income-10 (61%)  High-income- 11 (15%) | Low-income-7 (17%)  Middle-income-9 (59%)  High-income-12 (25%) | Low-income-6 (18%)  Middle-income-9 (60%)  High-income-11 (22%) | Use of efficient appliances  In different income groups | A  A  A | 3  7  2 | 2  6  3 | 2  7  3 |
|  | Electric fan | 68% | 61% | 53% | Use of consumption | A | 7 | 7 | 6 |
|  | Electric/gas heater | 27% | 39% | 39% | Hydro based | A | 3 | 4 | 4 |
|  | Vacuum cleaner | 28% | 41% | 36% |  | A | 3 | 5 | 4 |
| **Income and energy share in urban households** | Income and share of energy cost proportion | Low-income-13%  Middle-income-7%  High-income-4% | Low-income-14%  Middle-income-7%  High-income-4% | Low-income-11%  Middle-income-6%  High-income-3% |  | A  A  B | 2  1  10 | 2  1  10 | 2  1  10 |

Annex 1.2. Social sustainability indicator and checklist

| **Criteria** | **Sub-criteria** | **Inner-city** | **Middle-city** | **Outer-city** | **Description**  **(reason of value)** | **Values** | **Inner-city** | **Middle-city** | **Outer-city** |
| --- | --- | --- | --- | --- | --- | --- | --- | --- | --- |
| **Culture** | Context - Energy-intensive activities | 30 (Nos) | 12 (Nos) | 10 (Nos) | Increases excess fuel use | B | 4 | 8 | 8 |
| **Gender Participation** | Aspiration of new technology | 50% | 41% | 44% | Moving towards cleaner fuel | A | 6 | 5 | 5 |
| **Aspiration**  **Energy use in crisis**  **The tendency of shifting technology** | Crisis management (extra cylinders) | 22% | 20% | 20% | Towards fuel stacking – unsustainable | B | 8 | 9 | 9 |
|  | Crisis management (Induction) | 6% | 5% | 5% | Clean cooking (encouraging stage) | A | 6 | 5 | 5 |
|  | Crisis management (LPG + induction) | 2% | 4% | 3% | Towards cleaner cooking (percentage is small so taken as usual) | Small. | 2 | 4 | 3 |
|  | Crisis management (Kerosene +LPG) | 3% | 2% | 1% | Fossil fuel-based |  | 3 | 2 | 1 |
|  | Crisis management (fuelwood) | 4% | 5% | 5% | Managing household perspective –free source) | A | 1 | 1 | 1 |
| **Knowledge/ Perception** | Knowledge of Rainwater Harvesting | 15% | 22% | 24% | Sustainable behavior- saving | A | 2 | 3 | 3 |
| **Gender Participation on Activities** | Female Participation in CFP | 27% | 33% | 37% | Gender involvement | A | 3 | 4 | 4 |
|  | Female Participation in EAP | 18% | 12% | 16% | Gender involvement | A | 2 | 2 | 2 |
|  | Joint Participation CFP | 43% | 46% | 38% | Companion – sustainable approach | A | 5 | 5 | 4 |
|  | joint Participation EAP | 47% | 67% | 65% | Companion- sustainable approach | A | 5 | 7 | 7 |

Annex 1.3. Environmental sustainability indicator and checklist

| **Environmental context of energy consumption** | | | | | **Description** | **Values** | **Inner-city** | **Middle-city** | **Outer-city** |
| --- | --- | --- | --- | --- | --- | --- | --- | --- | --- |
| **Material use – quantity of appliances used** | Use of electric kitchen hoods |  |  |  |  |  |  |  |  |
|  | Exhaust fan | 21% | 41% | 38% | Exhaust fan | A | 3 | 5 | 4 |
|  | Chimney | 17% | 49% | 34% | Chimney | A | 2 | 5 | 4 |
| **Energy use- quantity and source of energy**  **Energy using model** | Energy use (LPG + induction) | 3% | 5% | 7% | Energy use (LPG) |  | 3 | 5 | 7 |
|  | Energy use (LPG) | 82% | 85% | 82% | Clean fuel | A | 9 | 9 | 9 |
|  | Gender role in kitchen work (Joint) | 25% | 39% | 36% | Joint work as sustainable | A | 3 | 4 | 4 |
|  | Cooking culture | 73% | 62% | 63% | Cooking more | B | 3 | 4 | 4 |
| **Indoor/outdoor Pollution- health** | No. of Windows (two windows) | 3% | 14% | 9% | No. of Windows (two windows) | A | 1 | 2 | 1 |
|  | Comfort during cooking -gender | 31% | 36% | 33% | Comfort feeling during cooking | A | 4 | 4 | 4 |
|  | Kitchen design inappropriate | 24% | 33% | 37% | Kitchen design | B | 8 | 7 | 7 |
|  | Air quality of kitchen (CO2) ppm (Max.) |  |  |  | Higher the PPM – health consequences | B | 3 | 6 | 4 |

| **Percentage (%)** | **Value A** | **Value B** |
| --- | --- | --- |
| 0-10 | 1 | 10 |
| 10 to 20 | 2 | 9 |
| 20 to 30 | 3 | 8 |
| 30 to 40 | 4 | 7 |
| 40 to 50 | 5 | 6 |
| 50 to 60 | 6 | 5 |
| 60 to 70 | 7 | 4 |
| 70 to 80 | 8 | 3 |
| 80 to 90 | 9 | 2 |
| 90 to 100 | 10 | 1 |

Annex 1.4. The rating system used for evaluating sustainability in local context
